# Lymphoid EVA1 Expression Is Required for DN1-DN3 Thymocytes Transition

**DOI:** 10.1371/journal.pone.0007586

**Published:** 2009-10-23

**Authors:** Stefano Iacovelli, Ilaria Iosue, Silvia Di Cesare, Maria Guttinger

**Affiliations:** 1 San Raffaele Biomedical Science Park Foundation, Rome, Italy; 2 Animal Technology Station, University of Rome Tor Vergata, Rome, Italy; New York University School of Medicine, United States of America

## Abstract

**Background:**

Thymus organogenesis and T lymphocyte development are accomplished together during fetal life. Proper development and maintenance of thymus architecture depend on signals generated by a sustained crosstalk between developing thymocytes and stromal elements. Any maturation impairment occurring in either cellular component leads to an aberrant thymic development. Gene expression occurring during T lymphocyte differentiation must be coordinated in a spatio-temporal fashion; one way in which this is achieved is through the regulation by cell-cell adhesion and interactions.

**Principal Findings:**

We examined the role played by Epithelial V-like Antigen 1 (EVA1), an Ig adhesion molecule expressed on thymus epithelial cells (TEC) and immature thymocytes, in T cell development by employing RNA interference *in vitro* and *in vivo* models. Fetal liver derived haematopoietic progenitors depleted of *Eva1*, displayed a delayed DN1-DN3 transition and failed to generate CD4CD8 double positive T cells in OP9-DL1 coculture system. In addition, we could observe a coordinated *Eva1* up-regulation in stromal and haematopoietic cells in coculture control experiments, suggesting a possible EVA1 involvement in TEC-haematopoietic cells crosstalk mechanisms. Similarly, *Rag2-γc* double knock out mice, transplanted with *Eva1* depleted haematopoietic progenitors displayed a 10-fold reduction in thymus reconstitution and a time delayed thymocytes maturation compared to controls.

**Conclusions:**

Our findings show that modulation of *Eva1* expression in thymocytes is crucial for lymphocyte physiological developmental progression and stromal differentiation.

## Introduction

The thymus is the primary lymphoid organ where T cells are generated [1] and, unlike the bone marrow (BM), it does not contain self-renewing progenitors [2]. Lymphopoiesis depends on the input of new progenitors from either BM in the adulthood or the fetal liver in the embryo [3] and thymic environment is required for differentiation into T lymphocytes [4]. Direct contact of haematopoietic precursor cells with thymic epithelial cells (TEC) allows the initiation of signalling events, that are relevant to the development of either cell types. In particular, lympho-stromal interactions are necessary for thymocyte development, thymus homeostasis and proper selection of immunocompetent T cell repertoire [5]. Interactions between TEC and developing thymocytes, named “thymic crosstalk” [6], are fundamental for the development of a normal thymic architecture [7], [8], [9].

T cell progenitors that seed the thymus do not express CD4 or CD8 and are commonly described as double-negative (DN) thymocytes. Based on CD44 and CD25 expression, they are further divided into four distinct DN subsets: CD44^+^CD25^−^ (DN1); CD44^+^CD25^+^ (DN2); CD44^−^CD25^+^ (DN3) and finally CD44^−^CD25^−^ (DN4) population [10]. Following DN stages, first CD4^+^CD8^+^ double positive (DP) thymocytes and finally mature CD4^+^ or CD8^+^ single positive (SP) lymphocytes are generated.

Epithelial V-like Antigen1 (EVA1) is an immunoglobulin-like, epithelium-specific, adhesion molecule expressed early in ontogeny [11]. It is strongly expressed in the emerging thymus, where it is down-regulated co-temporally with the emergence of the first DP thymocytes, [12]. In thymus EVA1 is expressed in both epithelial cells and developing DN thymocytes [13]. Transgenic overexpression of EVA1 in the thymus cortex resulted in a modified stromal environment, which elicited an increased absolute number of heterotypic complexes as thymic nurse cells (TNC), that are thought to be sites of lymphostromal interactions. The effect of EVA1 ectopic expression on the stromal microenvironment, together with the observation that stroma/haematopoietic ratio did not change, suggested that the increase in both cellularity and organ dimension was the result of an enhanced early stroma-thymocyte cross-talk.

Here we looked at the role of EVA1 in early T cell development by using fetal liver (FL) derived haematopoietic EVA1 depleted precursors for both *in vitro* and *in vivo* models. In OP9-DL1 coculture system [14], we observed *Eva1* up-regulation in haematopoietic cells during DN1-DN3 transition and a coordinated up-regulation in OP9-DL1 stromal cells. Conversely, *Eva1* interference caused a delay in DN1-DN3 transition and no up-regulation either in haematopoietic or in OP9-DL1 stromal cells, was observed. Similar *Eva1* depleted haematopoietic precursors were used in thymic repopulation experiments in alymphoid *Rag2-γc* double knock out (dKO) mice [15]. These mice reconstituted with *Eva1* interfered haematopoietic cells, showed a strong reduction in thymic reconstitution compared with controls, and a delay of DN1-DN3 progression with a consequent delay of thymocytes maturation.

Collectively our findings suggest that EVA1 plays a key role in the early phases of T cell development and that coordinated *Eva1* expression is necessary so that DN1-DN3 progression takes place correctly, suggesting a possible role in TEC-haematopoietic cells crosstalk.

## Results and Discussion

### Eva1 expression is finely regulated during thymus development in intrathymic haematopoietic cells at DN1-DN3 transition

**Figure 1:**
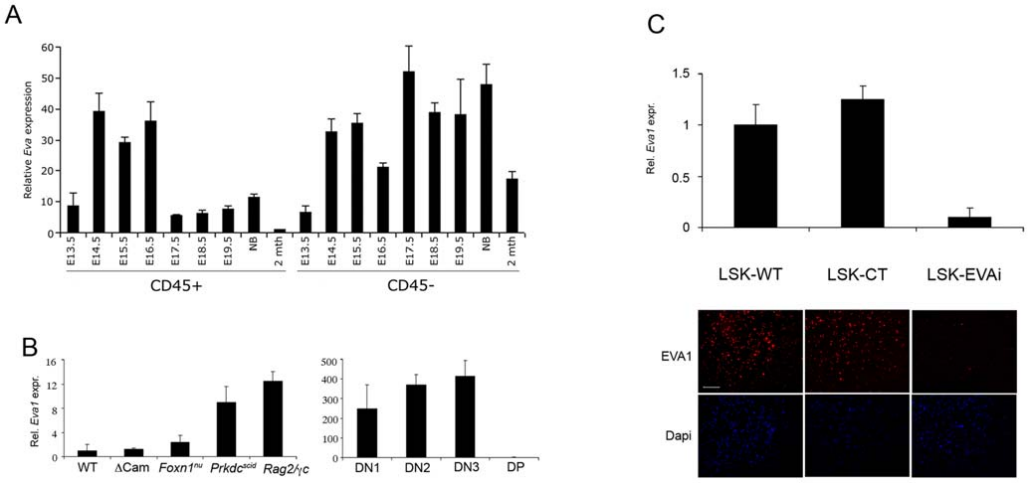
Analysis of *Eva1* expression. *Eva1* real-time RT-PCR in fetal thymi (A), adult DN subpopulations (B, left panel) and thymocytes from mutant mice (B, right panel). mRNA from flow cytometrically purified TECs (CD45-) and intrathymic haematopoietic cells (CD45+) or DN1-3 subpopulations was reverse transcribed and used as the template for PCR with *Eva1*-specific primers. All samples were normalized to the geometric mean of the GAPDH housekeeping gene. NB, newborn; 2mth, two months; WT, wild type; ΔCAM, Tg-Calcineurin; *Prkdc* ^scid^, protein kinase, DNA-activated, catalytic polypeptide (C, upper panel) Eva1 interference in LSK cells by lentiviral vector was controlled by real time RT-PCR. LSK-CT cells shown a comparable expression of *Eva1* while LSK-EVAi cells shown a drastic decrease of *Eva1* expression, indicating that the interference has occurred. (C, lower panel), fluorescence microscopy analysis confirming *Eva1* interference in LSK-EVAi cells. LSK-WT, uninfected LSK; LSK-CT, LSK infected with a non-interfering lentiviral vector; LSK-EVAi, LSK infected with a *Eva1*-interfering lentiviral vector.

In a previous work aimed at defining the cell lineages expressing EVA1 during thymus organogenesis [13], we demonstrated that EVA1 is expressed in both epithelial and haematopoietic cells. While expression in TEC appeared to be stable, the one in haematopoietic cells was specific for DN subsets [13]. To characterize timing of *Eva1* expression during thymus ontogeny in the different cellular components, quantitative RT-PCR (qRT-PCR) was performed in flow cytometry purified haematopoietic cells (CD45^+^) and TEC (CD45^−^), from wild type mice at different stages of embryonic development (from embryonic day 13.5–E13.5 to newborn - NB). As shown in figure 1A, in CD45^+^ cells, *Eva1* showed an expression peak between E14.5 and E16.5 stages, then was strongly downregulated. *Eva1* modulation was probably related to DN subsets appearance and progression toward later stages of development, such as DP cells. We also confirmed that, in TEC, *Eva1* expression level was stable from E13.5 to birth, then was moderately downregulated to a steady state which was maintained throughout adult life (Fig. 1A) [13]. Notably, a slight increase of *Eva1* expression could be observed at the stage immediately after the downregulation seen in the haematopoietic counterpart (i.e., E17.5) (Fig. 1A), suggesting a coordinated EVA1 modulation between TEC and developing thymocytes.

We then used mutant mice with an early block in thymus development to define EVA1 requiring stages. We observed a marked increase of *Eva1* expression in sorted CD45^+^ thymocytes from SCID (with a spontaneous mutation in protein kinase, DNA-activated, catalytic polypeptide *Prkdc* ^scid^,) and *Rag2-γc* dKO mice, but not from *Foxn1* KO nude or Tg-calcineurin mice (Δ*Cam*) (Fig. 1B left panel). Interestingly, in both SCID and *Rag2-γc* dKO thymi, the majority of CD45+ cells are DN1-DN3 stage cells [15], [16]. In contrast, *Foxn1* ko nude mice have a pre-DN cells block [17], [18], [19] and Tg-calcineurin mice express a constitutively active form of calcineurin, have an arrest of thymocyte development around DN4 stage [20].

Tydell et al., using mutant mice, demonstrated that also adult DN subsets such as T cell precursors sorted cells express *Eva1* [21]. To verify that the above observation was not mutation-dependent, we looked at *Eva1* expression in sorted DN subpopulations from two-month-old C57Bl6 thymi. As shown in Figure 1B (right panel), we indeed observed up-regulation of *Eva1* during DN1-DN3 transition, with an expression peak at DN3 stage and a sharp decline at DP stage, confirming that *Eva1* positive regulation takes place during adult DN1-DN3 transition, too.

All together these data confirm that *Eva1* is transcriptionally regulated during DN1-DN3 transition.

### LSK-EVAi cells had a delayed DN1-DN3 transition and failed to generate CD4 and CD8 SP cells in in vitro T cell development

**Figure 2:**
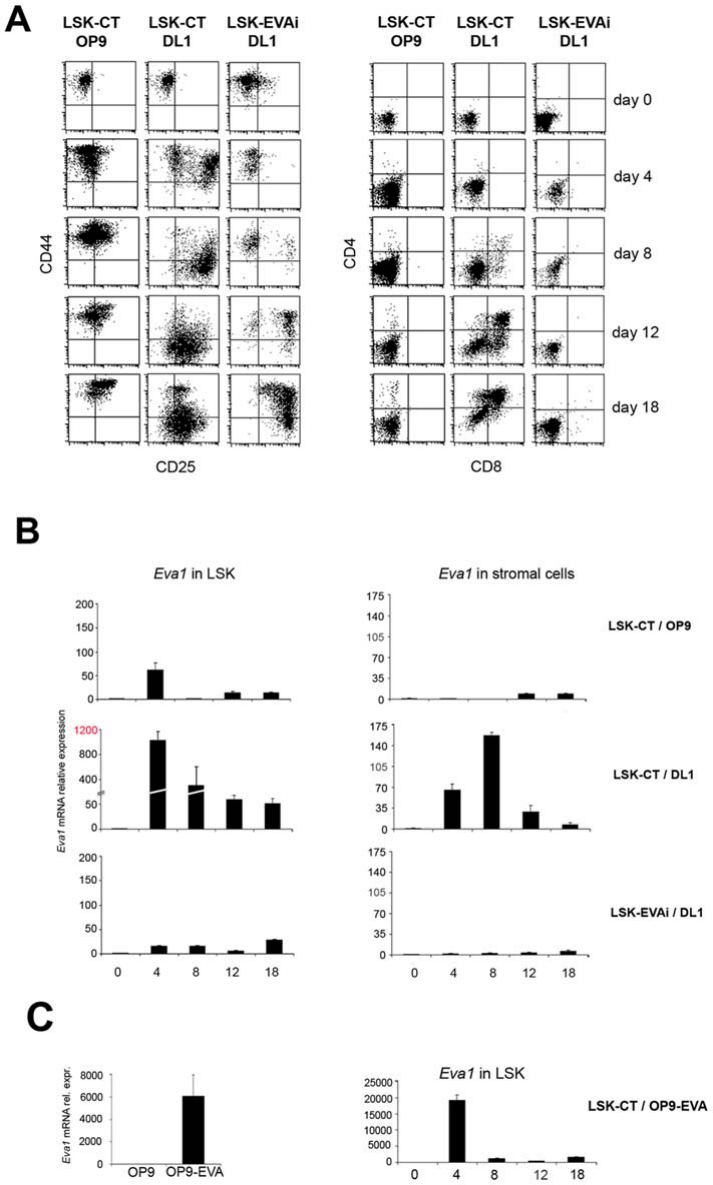
*In vitro* T cell development with LSK-EVAi cells. (A) Time course of T cell differentiation of LSK cells infected with a non-interfering (LSK-CT) or with an Eva1 interfering lentivirus (LSK-EVAi) respectively, cocultured with either OP9-GFP control cells or OP9-DL1. Flow cytometric analysis of CD25/CD44 (left panel) and CD4/CD8 (right panel) stainings revealed that LSK-EVAi cells had a delayed DN1-DN3 transition and failed to generate CD4 and CD8 cells in OP9-DL1 cocultures. (B) Time course of Eva1 expression in both haematopoietic (left panels) and stromal (right panels) cells, respectively. Cocolture combinations are indicated. No variations in EVA1 expression are observed in either cell type, from cocoltures that do not sustain T cell development (LSK-CT/OP9 and LSK-EVAi/DL1). Eva1 is strongly up-regulated at 4 and 8 days of LSK-CT/DL1 coculture, during DN1-DN3 transition. Note: scale amplitude in Eva1 real-time RT-PCR in haematopoietic cells. Eva1 is concomitantly up-regulated in stromal OP9-DL1 counterpart too. (C) Right panel: time course of Eva1 expression in haematopoietic cells coltured on OP9 cells overexpressing EVA1. Left panel: EVA1 expression in OP9 and OP9-EVA cells, respectively.

T lymphocyte development, can be studied *in vitro* by coculturing haematopoietic progenitors with OP9 stromal cells expressing Notch ligand Delta-like-1 (OP9-DL1). We applied this system together with *Eva1* lentiviral RNA interference (RNAi) to evaluate the role of EVA1 in T cell differentiation [14]. Haematopoietic precursors isolated from E14,5 fetal liver (FL) by cell sorting (Lin^−^; Sca1^+^; c-Kit^+^)(LSK) were used. Interference with *Eva1* was performed by infection with lentiviral vectors (see material and methods for details). The efficiency of RNA interference was assessed by qRT-PCR and immunofluorescence analysis (IF). As shown in Figure 1C (upper panel), LSK cells interfered for *Eva1* (EVAi) showed a strong reduction of *Eva1* expression, while LSK infected with control lentiviral vector (CT) showed an expression level of *Eva1* comparable to uninfected LSK-WT cells. Interference was confirmed by IF (Fig. 1C, lower panel) using a rabbit polyclonal serum anti-EVA1. We tested three different interfering sequences obtaining comparable results (data not shown). To address whether EVA1 depletion had an effect on T cell development, progressive phenotype acquisition based on CD44/CD25 and CD4/CD8 expression, was evaluated by flow cytometry in cocultures experiments. As described [14], LSK-CT cocultured with OP9-GFP cells did not show the CD44/CD25 progression and did not give rise to T cells (Fig. 2A, left and right panels). Otherwise, after 4 days of coculture, LSK-CT cells cultured on OP9-DL1 cells showed a differential surface expression of CD44/CD25 molecules (Fig. 2A, left panel). The progression in CD44/CD25 maturation pathway was observed until 18 days of coculture (Fig. 2A, left panel). This progression correspond to CD4 and CD8 expression (Fig. 2A, right panel). LSK-CT cells gave rise to CD4^+^CD8^+^ immature double positive (DP) T cells after 8 days of coculture (Fig. 2A, right panel), with an increase of DP cells after 18 days of coculturing (Fig. 2A, right panel). The temporal kinetics of LSK-CT differentiation cocultured with OP9-DL1 cells was similar to that observed with LSK-WT cells (data not shown). A delayed progression into the CD44/CD25 maturation pathway was observed when LSK-EVAi cells were cultured on OP9-DL1 cells and compared to LSK-CT progression (Fig. 2A, left panel). After 18 days, LSK-EVAi cells were arrested at DN2-DN3 stages and failed to generate DP cells (Fig. 2A, right panel). These data confirmed that *Eva1* upregulation occurs at DN1-DN3 transition during physiological thymocyte development and maturation *in vitro*.

Furthermore, we looked at *Eva1* regulation in LSK and stromal cells, during *in vitro* differentiation. qRT-PCR analysis showed that no *Eva1* regulation was detectable in both cellular partners of negative control cocultures LSK-CT/OP9 (Fig. 2B, upper panels), while *Eva1* expression was increased in LSK-CT cells at day 4 and 8 of coculture, with a peak on day 4, corresponding to the DN1-DN3 transition (Fig. 2B, centre panels). *Eva1* upregulation, was also observed in stromal cells with a peak at day 8, in agreement with data obtained on *ex-vivo* cells (Fig. 1A). The great early lymphoid *Eva1* upregulation observed in the positive control cocultures, seemed to be driven by the contact with the stromal partners and accompained physiological differentiation steps, while no variations in *Eva1* expression levels were observed in LSK-EVAi/DL1 (Fig. 2B, lower panels). Sequential modulation in the two cellular partners underlines the role of EVA1 in thymocyte-stroma cross-talk (Fig. 2B, centre panels), demonstrating that EVA1 expression is required for DN1-DN3 transition and that its lymphoid positive regulation needs haematopietic/stromal interactions. Furthermore, to define if EVA1 expression in stromal cells was sufficient for DN differentiation, we expressed EVA1 in OP9 cells. Coculture experiments with LSK-CT and OP9-EVA cells did not elicited T cell differentiation and the tremendous increase (twenty thousand fold) of *Eva1* expression after four days of coculture (Fig. 2C) was not enough to generate DN cells. These results indicated that EVA1 expression *per se* is not sufficient to drive early T cell maturation and needs other molecules/signal derived by Notch/Delta-ligand-1 interactions.

### Thymus reduction in Rag2-γc double knockout mice reconstituted with LSK-EVAi

**Figure 3:**
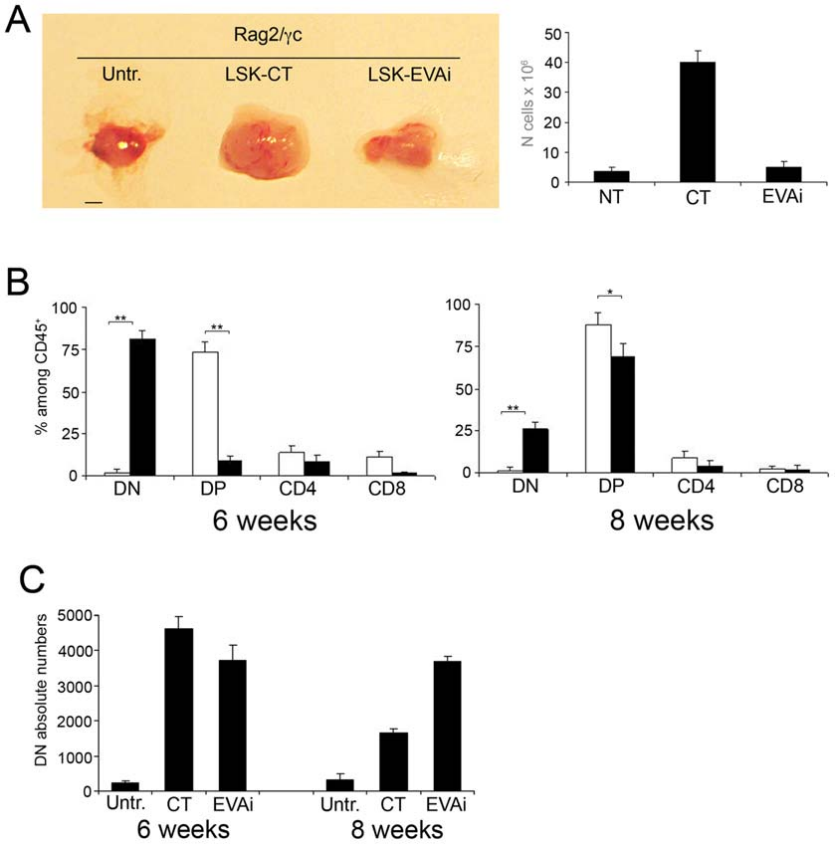
*In vivo* T cell development with LSK-EVAi cells. (A, left panel) *Rag2/γc* mice were transplanted with either LSK cells infected with a non-interfering (LSK-CT) or with an Eva1-interfering lentivirus (LSK-EVAi). After 8 weeks, thymi were excised and compared: thymus from mice transplanted with LSK-EVAi were comparable to untreated ones for dimension and total cellularity (A, right panel). Untr., untreated control animal. Scale bar, 2 mm. (B) Time course of T cell development of LSK-CT (white bars) or LSK-EVAi (black bars) cells respectively, in recostitution experiments in *Rag2/γc* mice. Flow cytometric analysis of CD25/CD44 and CD4/CD8 stainings revealed that LSK-EVAi reconstituted thymus had a delay of T cell differentiation with an accumulation of DN cells at 6 weeks after trasplant (left panel). Percentage of DN cells in LSK-EVAi reconstituted thymus persists higher than in LSK-CT reconstituted thymi at 8 weeks after transplant (right panel). Mean and SD of three independent experiments are shown (** p<0.01). (C) Counts of total DN cells generated in the three different mouse groups at six and eight weeks post-treatment.

T cell development in the thymus depends on the continuous recruitment of haematopoietic precursors from the bone marrow (BM) via the blood [3]. Mice with deletion of the common cytokine receptor *γ* chain (*γc*) and recombinase activating gene 2 (*Rag2-γc* dKO) have a stable phenotype characterized by the absence of all T lymphocytes, B lymphocytes and NK cells. We performed BM transplantation experiments to assess the ability of LSK-CT and LSK-EVAi cells to reconstitute a functional thymus in *Rag2-γc* dKO sublethally irradiated mice. After 8 weeks from the transplant, recipient mice injected with LSK-CT cells showed a marked thymic reconstitution if compared with not transplanted mice (Fig. 3A, left panel). Mice transplanted with LSK-EVAi cells showed a smaller thymus compared with the LSK-CT-injected mice, indicating a less efficient thymic reconstitution (Fig. 3A, left panel). This result was confirmed by total cell numbers that were significantly smaller in LSK-EVAi than in LSK-CT reconstituted mice (Fig. 3A, histogram). Similar results were obtained at 6 weeks after transplant (data not shown).

Reconstituted thymi were analyzed by flow cytometry 6 and 8 weeks after transplantations for CD44/CD25 and CD4/CD8 subsets distribution. Thymi from mice transplanted with LSK-CT cells displayed higher percentages of DP and SP cells compared with thymi from mice transplanted with LSK-EVAi cells (Fig. 3B); the difference in DP and SP subpopulations distribution between the two groups was still evident after 8 weeks (Fig. 3B). LSK-EVAi cells reconstituted thymi showed a delayed in cell differentiation, with accumulation of DN thymocytes already evident at 6 weeks from transplant (Fig. 3B). Thereafter, there was an increase of DP cells and the percentage of DN cells still remained significantly (p<0.01) higher than in LSK-CT reconstituted thymi (Fig. 3B). Comparison of DN counts obtained from the three different mouse groups, showed that spontaneous generation of DN subset was very modest even at eight weeks from irradiation, indicating that endogenous progenitors are not implicated in reconstitution events. These results confirm that EVA1 is important for normal developmental processes.

**Figure 4:**
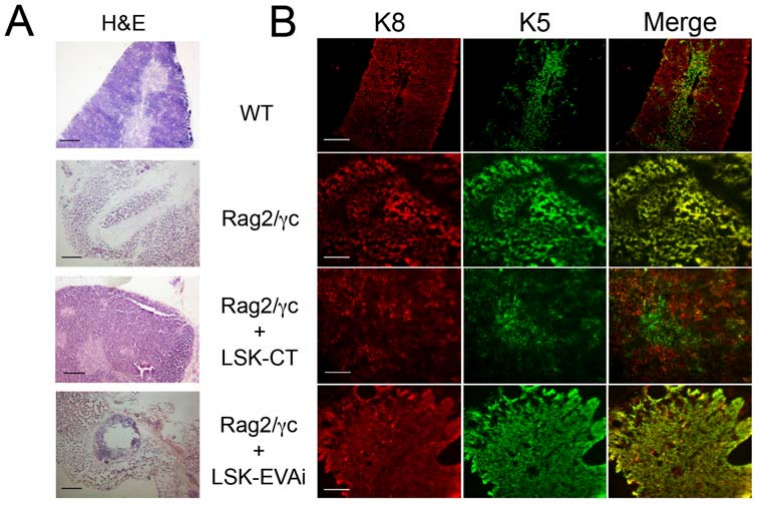
LSK-CT cells restore 3D thymus architecture in transplanted mice. (A) Hematoxylin-Eosine staining of thymi from *Rag2-γc* mice untreated or six weeks after transplantion with either LSK cells infected with a non-interfering (LSK-CT) or with an *Eva1*-interfering lentivirus (LSK-EVAi). Upper panel shows a normal one-month-old C57Bl6 (wt, wild type) thymus. Thymus from untreated ten-weeks-old *Rag2-γc* double KO mice shows an extremely low number of thymocytes and an aberrant morphology. A significant haematopoietic cells repopulation was observed in thymus from LSK-CT reconstituted mice. On the contrary, in thymus from LSK-EVAi transplanted mice only few haematopoietic cells repopulate the thymus. c: cortex; m: medulla. Scale bars, 200 µm; magnification 20X. (B) Fluorescence confocal microscopy analysis showing keratin (K) 5 and 8 distribution in C57Bl6 (WT, wild type), mice utreated or transplanted with either LSK cells infected with a non-interfering (LSK-CT) or with an Eva1-interfering lentivirus (LSK-EVAi). *Rag2-γc* thymus has an aberrant keratin distribution and 2-D thymus organization. LSK-CT thymus showed keratin redistribution and conversion in 3-D thymus organization otherwise absent in LSK-EVAi thymus. Scale bars 200 µm; magnification 20X.

Patterning of thymic epithelial compartment is defined by differential keratin (K) expression: in particular thymic cortex is characterized by the expression of K8 and K18, while thymic medulla expresses K5 and K14 isoforms. While initial patterning does not depend on inductive signals from haematopoietic cells, thymocyte derived signals are required during late phases of fetal thymus development to generate and sustain a normal thymic epithelial compartment in newborn and adult mice [22]. Moreover, thymic stroma consists of TEC organized in a three-dimensional (3-D) structure [23], which is driven by developing T cells via TEC crosstalk [6]. Haematoxylin and eosin staining of thymi from *Rag2-γc* dKO transgenic mice revealed thymocyte paucity (Fig. 4A), while immunohistochemistry showed an aberrant K8^+^K5^+^ phenotype (Fig. 4A, 4B), [24] and a 2-D thymus organization (Fig. 4B).(Fig. 4A). LSK-CT cells transplanted into *Rag2-γc* dKO mice led to redistribution of keratins and reappearance of 3-D thymic architecture (Fig. 4B). Moreover, haematoxylin and eosin analysis revealed that a significant amount of haematopoietic cells repopulated the thymus after transplantation (Fig. 4A). Conversely, in LSK-EVAi transplanted mice we observed only a partial reappearance of 3-D thymic architecture and modest keratin redistribution (Fig. 4B), with a few lymphocytes repopulating thymus (Fig. 4A). Possibly, the absence of EVA1 homotypic interactions leads to DN1-DN3 progression delay, which prevents stromal thymus reorganization. In addition we further show that DN1-DN3 transition controls thymic epithelium morphogenesis [25], [26].

Taken together, our results demonstrate that EVA1 is important in early T cell differentiation, affecting DN maturation and is a key molecule in thymocyte-stroma cross-talk.

## Materials and Methods

All experiments were approved by the Italian Health Ministry, according european legislation.

### Mice

C57/Bl-6 (Charles River, Calco-I) and C57/Bl-6 *Rag2-γc* dKO (Taconic Europe, DK) were maintained under specific pathogen free (SPF) conditions at Fondazione Parco Biomedico S. Raffaele, Rome. The day of first vaginal plug detection was designated as gestation day 0.5 (E0.5). All experiments were approved by the Italian Health Ministry, according european legislation.

### RNA extraction and quantitative RT-PCR

Total RNA was extracted from sorted cell populations using the RNeasy plus mini kit (Qiagen GmbH, D-40724 Hilden, Germany), and reverse transcribed with oligo-dT primer and superscript RT II reverse transcriptase (Invitrogen corporation, 92008 CA, USA). Real time PCR was performed on diluted cDNA samples using TaqMan Universal PCR Master Mix in ABI PRISM 7500 Real Time PCR System. All materials, including primers for EVA (EVA-FAM) and GAPDH standard (GAPDH-VIC) were purchased from Applied Biosystem (94404 CA, USA). Samples were examined in triplicate. The DELTA Ct method was used for normalization to GAPDH mRNA.

### Lentiviral production and infection

The lentiviral vector pLKO.1 carrying the sequence CGGACAGATGTTCGGTTAAA (EVAI 336) or GCGCTAACTGTGACGTGGAAT (EVAi-391) or CAGGTGAAGAATCCACCTGAT (EVAi-589) were used to produce the virus for interfering murine Eva RNA. As negative control was used a non-target shRNA vector carrying the sequence CAACAAGATGAAGAGCACCAA (SCR) (all mission shRNA plasmid DNA vectors were purchased by Sigma Life Science, St Louis, MO, USA). Lentiviral supernatants were generated into 293T cells by transient co-transfections, of lentiviral constructs and appropriate packaging vectors with Fugene6 (Roche Diagnostic corporation, Monza, Italy). After 48 hours supernatants were collected and employed to infect LSK cells (1×10^6^ cells/sample) in retronectin (Takara Bioinc Otsu, Shiga 520–2193, Japan) precoated (50 µg/ml) non-tissue culture 6-well plates (Falcon, BD, 92121 CA, USA). Supernatants were supplemented with 10 ng/ml IL3, 20 ng/ml IL6, 50 ng/ml SCF, 25 ng/ml IL7 (all cytokines from Immunotools, GmbH, 26169 Friesoythe, Germany) and added to cells seeded in 6-well plates. Infections proceeded for 24 hours at 37°C, with at least four changes of viral supernatants. Then cells were harvested, washed with PBS (Euroclone, 20016 Pero (MI), Italy) and replated in minimum essential medium alpha (alpha MEM) (Invitrogen) with 20% fetal bovine serum (FBS) (Hyclone, 84321 Utah, USA) and cytokines as above. After 48 hours, LSK cells were selected with puromycin (Sigma,) 2 µg/ml for additional 48 hours and used for coculture or transplant experiments.

### Flow Cytometry and cell sorting

Isolation and purication of heamatopoietic progenitors were performed as described [27]. Briefly, fetal liver (FL) cells obtained from embryonic day 14,5 (E14,5) C57/Bl-6 mouse embryos were depleted of Gr1^+^, CD49b^+^, CD11b^+^, CD3e^+^, CD19^+^, Ter119^+^ and TCRβ^+^ cells through labeling with the corresponding biotinylated antibodies (all eBioscience, 92212 CA, USA), followed by incubation with streptavidin coated magnetic beads (Miltenyi Biotec Inc, 95602 Auburn CA, USA). Labeled cells were subsequently removed by magnetic column separation (Miltenyi Biotec). Lineage-depleted (Lin^−^) FL cells were stained with c-Kit-PE and Sca1-FITC (eBioscience) and sorted on a FACS DiVa (BD Biosciences) and analyzed with Cell Quest software (BD Biosciences). After magnetic purification with CD45 MACS-microbeads (Miltenyi Biotec), cells were analyzed by FACScalibur (BD Biosciences) instrument. Cells were first incubated with normal mouse serum and CD16/CD32 Fc receptor (eBioscience) for blocking nonspecific antibody binding and finally incubated with anti-CD44 (eBioscience)/CD25 (eBioscience) or anti-CD4 (eBioscience)/CD8 (eBioscience). mABs were directly conjugated to PE or APC.

### Cocultures

OP9 coculture assays were performed as described [23]. Stromal cells were plated 48 hours before use in 24-well plates (Falcon, BD) to achieve a confluent monolayer. All cocultures were initiated with 4×10^3^FL Lin^−^Sca1^+^c-Kit^+^ (LSK) in alpha MEM-20% FBS in the presence of 5 ng/ml Flt3L and 5 ng/ml IL7 (Immunotools). Cocultures were harvest by forceful pipetting at the indicated time points. LSK were separated from stromal cells by labeling with CD45-coated magnetic beads (Miltenyi Biotec). Labeled cells were subsequently separated and recovered by magnetic column separation (Miltenyi Biotec).

### Immunohistochemistry

Thymuses for sectioning were washed in PBS, embedded in OCT compound (Bio-Optica, 20134 Milano, Italy) and snapped frozen. Five µm frozen sections were cut and plated onto Poly-l-lysine slides (Menzel-Glaser, Menzel GmbH, D-38116 Braunschweig, germany). For immunostaining, sections or cells were permeabilized in PBS-1%BSA 0.2%TritonX100 and blocked in 20% normal goat serum, then incubated with primary antibody for 1–2 hours followed by incubation with the appropriate secondary antibody. Immunoreaction was analyzed and photographed using fluorescent microscope (Nikon Eclipse TE 2000-E) and Metamorph 7.5 software.

### In vivo thymus reconstitution

5×10^5^infected and selected LSK cells were intravenously injected into irradiated *Rag2-γc* dKO(4 Gy) mice. Thymi were analyzed 6 or 8 weeks after transfer by flow cytometry. Donor cells were distinguished from host cells by their ability to differentiate in lymphoid cells.

### Statistics

Statistical comparison was performed by the Student's t-test using Excel software.
